# A Modified Two-Relaxation Thermoelastic Model for a Thermal Shock of Rotating Infinite Medium

**DOI:** 10.3390/ma15249056

**Published:** 2022-12-18

**Authors:** Maryam H. Aljadani, Ashraf M. Zenkour

**Affiliations:** 1 Department of Mathematics, Jamoum University College, Umm Al-Qura University, Makkah 21421, Saudi Arabia; 2 Department of Mathematics, Faculty of Science, King Abdulaziz University, Jeddah 21589, Saudi Arabia; 3 Department of Mathematics, Faculty of Science, Kafrelsheikh University, Kafrelsheikh 33516, Egypt

**Keywords:** Lord–Shulman theory, Green–Lindsay theory, thermal shock, rotating half-space

## Abstract

A unified form of thermoelasticity theory that contains three familiar generalized thermoelasticity. The Lord–Shulman theory, Green–Lindsay theory, and the classical one can be outlined in this form. The field quantities of a rotating/non-rotating half-space with and without the effect of the decay parameter can be obtained due to the unified thermoelasticity theory. The present medium is subjected to a time-dependent thermal shock taking into account that the magnitude of the thermal shock wave is not totally fixed but decaying over time. A special case of a thermal shock waveform with constant magnitude may be considered. The field quantities such as temperature, displacements, and stresses of the present problem are analytically obtained. Some plots of these field variables are presented in two- and three-dimensional illustrations in the context of refined theories.

## 1. Introduction

Thermoelasticity theory, which involves the influence of temperature change, is well established. The temperature field is associated with the elastic strain field, based on the theory. Fourier’s conduction equation is widely utilized in numerous engineering applications in thermoelasticity or classical heat transmission. In conjunction with the strain-rate term to the Fourier heat conduction formula, Biot’s [1] presented the classical coupled thermoelasticity theory that gives a parabolic heat conduction formula known as the diffusion equation. This theory suggests that elastic waves have a finite propagation speed whereas thermal shocks have an infinite speed. Physically, this cannot be carried out. To address this issue, Lord and Shulman (LS) [2], and Green and Lindsay (GL) [3], established generalized thermoelasticity theories proposing the presence of finite thermal wave speed in the structures. The two generalized theories suggested one or two relaxation times in the thermoelastic interaction, which adjusted Fourier’s heat conduction formula to determine a finite speed for thermal wave propagation, which was adjusted by correcting the Neumann-Duhamel equation and the energy formula. As a result of modifications introduced to the generalized two theories, heat propagation was shown as a wave phenomenon instead of a diffusion one, which is generally related to the influence of the second sound. Since the structures of these two theories differ from one another, they cannot be gotten as a unique case of one to the other. The goal of developing generalized thermoelasticity theories was designed to address the problem of infinite heat propagation present in the classical coupled dynamical thermoelasticity theory.

Researchers used generalized thermoelastic models to uncover different unique thermoelastic behaviors with additional external factors. Othman and Mondal [4] investigated the LS model of 2D plane waves in a generalized thermoelastic medium considering memory-dependent derivatives. Under LS’s single relaxation time theory, the generalized thermoelasticity behavior of a rotating disk consisting of homogeneous and isotropic material is addressed by Kiani [5]. Youssef et al. [6] used the hyperbolic two-temperature generalized thermoelasticity theory to investigate the effect of rotation on a thermoelastic solid sphere. Sadeghi and Kiani [7] discussed the generalized magneto-thermoelastic behavior of a layer utilizing LS and GL’s theories. Tzou [8] proposed a dual-phase-lag (DPL) delay model of heat conduction in acroscopic design to incorporate the impacts of infinitesimal interactions in the fast-transient method of heat transportation mechanism. Heydarpour et al. [9] applied the LS thermoelasticity theory to investigate the transient thermoelastic response of functionally graded graphene platelets reinforced composite (FG-GPLRC) spherical shells subjected to thermomechanical loadings. Using the DPL and LS models, Abd-Alla et al. [10] investigated the influence of an electromagnetic field, gravity field, rotation, and initial stress on a generalized thermoelastic porous material. By developing the Roychoudhuri model, Abouelregal [11] provided a modified thermoelastic model of heat conduction with higher-order time derivatives. Zenkour et al. [12] used a refined multi-time-derivative DPL thermoelasticity and LS models to investigate the magneto-thermoelastic behavior in an infinite medium including a spherical cavity. In the framework of LS formulation, Alibeigloo [13,14] discussed the time-dependent behavior of a sandwich plate with a functionally graded core as well as the thermal shock problem of a simply supported carbon nanotube-reinforced composite rectangular plate.

The investigations of additional issues in thermoelastic rotating media with various thermoelasticity theories are found in [15,16,17,18,19,20]. Using the finite element approach, Othman and Abbas [21] investigated the influence of plane wave rotation at the free surface of a fiber-reinforced thermoelastic half-space. Moreover, several cases of thermoelastic problems considering thermal shock using different thermoelasticity theories are investigated. Zenkour [22] discussed the three-dimensional thermal shock plate problem by comparing several generalized thermoelasticity models. Othman and Song [23] used distinct generalized thermoelasticity models to investigate the electro-magneto-thermoelastic interactions in a semi-infinite entirely conducting body exposed to a thermal shock to the surface. Tehrani and Eslami [24] applied the LS and GL models to discuss the boundary element analysis in the finite domain. Abouelregal et al. [25] solved the Moore–Gibson–Thompson equation of an unbounded medium with a cylindrical hole. For more details, one can refer to the review of Shakeriaski et al. [26] concerning the recent advances in generalized thermoelasticity theory and the modified models.

Many investigators are interested in the thermoelastic problems of rotating infinite mediums under thermal shock. Xiong and Tian [27] investigated the transient effects of a rotating electromagnetic-thermoelastic half-space with porosity and diffusion under thermal shock using generalized thermoelasticity without energy dissipation. Sharifi [28] applied the LS-coupled thermoelasticity theory to address the thermal shock issue in an orthotropic spinning disk. Mashat et al. [29] proposed a novel model of generalized thermoelasticity formulas for a rotating infinite orthotropic structure with a cylindrical hole under thermal shock. Abbas and Zenkour [30] applied Green–Naghdi (GN) model to examine the impact of initial stress and rotation on the thermal shock problem of a fiber-reinforced anisotropic half-space. Lotfy and Hassan [31] examined the impact of rotation on a two-temperature generalized thermoelasticity model exposed to thermal shock. Abouelregal and Marin [32] used the LS theory for the size-dependent thermoelastic vibrations of nanobeams.

A unified form of refined thermoelasticity theories is presented in this article. Two familiar generalized thermoelasticity theories are based on Lord and Shulman, and Green and Lindsay’s theories. The field quantities of a rotating/non-rotating half-space with and without the effect of the decay parameter are obtained. The medium is exposed to a time-dependent thermal shock. The displacements, temperature, and stresses of the present problem are analytically obtained and discussed.

## 2. Basic Governing Equations

In this section, we will examine a rotating thermoelastic half-space (z≥0). The plane wave propagation problem across x-axis will be addressed. The current half-space is initially at rest, and the surface z=0 is traction-free. The surface experiences a time-dependent thermal shock. As a result, all field values studied will be functions of time as well as the coordinates x and z.

The components of displacement $u_{i}$ are written as follows:

(1) $$u_{1}=u\left(x,z,t\right),\;\;\;u_{2}=0,\;\;\;u_{3}=w\left(x,z,t\right).$$

The heat conduction formula is given as:

(2) $$K\nabla^{2}\theta =\rho c_{E}D_{1}\frac{\partial \theta }{\partial t}+\gamma T_{0}D_{1}^{m}\frac{\partial e}{\partial t},$$

where $\nabla^{2}=\frac{\partial^{2}}{\partial x^{2}}+\frac{\partial^{2}}{\partial z^{2}}$; $\theta =T-T_{0}$, wherein T is the temperature over the reference temperature $T_{0}$; ρ is the medium density; $c_{e}$ is the certain heat at constant strain, *K* is the parameter of thermal conductivity; $e=e_{kk}=u_{k,k}$ is the volumetric strain where *u_i_* is the components of displacement; $\gamma =\alpha_{t}\left(3\lambda +2\mu \right)$ is the parameter of thermoelastic coupling in which λ, μ are Lame’s constants and $\alpha_{t}$ is the coefficient of thermal expansion; and $D_{1}$, $D_{1}^{m}$ are the operators of time-derivative, which can be written as [33,34,35,36,37,38,39,40],

(3) $$D_{1}=1+\sum_{n=1}^{N}\tau_{1}^{n}\frac{\partial^{n}}{\partial t^{n}},\;\;\;D_{1}^{m}=1+\delta_{1m}\sum_{n=1}^{N}\tau_{1}^{n}\frac{\partial^{n}}{\partial t^{n}},\;\;\;N\geq 1,$$

where $\tau_{1}$ and *δ*_1*m*_ indicate the first relaxation time and Kronecker’s delta, respectively.

The constitutive equations according to the generalized thermoelasticity theory can be written as,

(4) $$\sigma_{ij}=\left(\lambda e-\gamma D_{2}^{m}\theta \right)\delta_{ij}+2\mu e_{ij},$$

where $\sigma_{ij}$, $e_{ij}$ and $\delta_{ij}$ represent the stress tensor, strain tensor, and Kronecker’s delta, respectively, and $D_{2}^{m}$ indicates a time-derivative operator which is presented by,

(5) $$D_{2}^{m}=1+\delta_{2m}\sum_{n=1}^{N}\tau_{2}^{n}\frac{\partial^{n}}{\partial t^{n}},\;\;\;N\geq 1,$$

wherein $\tau_{2}$ and $\;\delta_{2m}$ are the second relaxation time and Kronecker’s delta.

The strain elements can be written as the displacement functions $u_{i}$

(6) $$e_{ij}=\frac{1}{2}\left(u_{i,j}+u_{j,i}\right).$$

Subsequently, Equation (4) becomes,

(7) $$\sigma_{xx}=\left(\lambda +2\mu \right)\frac{\partial u}{\partial x}+\lambda \frac{\partial w}{\partial z}-\gamma D_{2}^{m}\theta ,$$

(8) $$\sigma_{zz}=\left(\lambda +2\mu \right)\frac{\partial w}{\partial z}+\lambda \frac{\partial u}{\partial x}-\gamma D_{2}^{m}\theta ,$$

(9) $$\sigma_{xz}=\mu \left(\frac{\partial w}{\partial x}+\frac{\partial u}{\partial z}\right).$$

In the presence of rotation, the two-dimensional (x,z) model of equations of motion for a thermoelastic medium can be derived as,

(10) $$\left(\lambda +2\mu \right)\frac{\partial^{2}u}{\partial x^{2}}+\mu \frac{\partial^{2}u}{\partial z^{2}}+\left(\lambda +\mu \right)\frac{\partial^{2}w}{\partial x\partial z}-\gamma D_{2}^{m}\frac{\partial \theta }{\partial x}=\rho (\frac{\partial^{2}u}{\partial t^{2}}-\Omega^{2}u+2\Omega \frac{\partial w}{\partial t}),$$

(11) $$\left(\lambda +2\mu \right)\frac{\partial^{2}w}{\partial z^{2}}+\mu \frac{\partial^{2}w}{\partial x^{2}}+\left(\lambda +\mu \right)\frac{\partial^{2}u}{\partial x\partial z}-\gamma D_{2}^{m}\frac{\partial \theta }{\partial z}=\rho (\frac{\partial^{2}w}{\partial t^{2}}-\Omega^{2}w-2\Omega \frac{\partial u}{\partial t}).$$

## 3. Formulation of the Problem

The following non-dimensional variables can be used to modify the previous equations into non-dimensional forms:

(12) $$\begin{array}{c} \left\{x^{\prime },z^{\prime },u^{\prime },w^{\prime }\right\}=\frac{c_{0}}{\eta }\left\{x,z,u,w\right\},\;\;\;\left\{t^{\prime },\tau_{1}^{\prime },\tau_{2}^{\prime }\right\}=\frac{c_{0}^{2}}{\eta }\left\{t,\tau_{1},\tau_{2}\right\},\;\;\;\theta^{\prime }=\frac{\gamma \theta }{\rho c_{0}^{2}},\;\\ \sigma_{ij}^{\prime }=\frac{\sigma_{ij}}{\rho c_{0}^{2}},\;\;\;\Omega^{\prime }=\frac{\eta }{c_{0}^{2}}\Omega ,\;\;\;\eta =\frac{K}{\rho c_{E}},\;\;\;c_{0}=\sqrt{\frac{\lambda +2\mu }{\rho }}. \end{array}$$

As a result, the non-dimensional stress forms may be represented as (for convenience, the primes are omitted),

(13) $$\sigma_{xx}=\frac{\partial u}{\partial x}+c_{1}\frac{\partial w}{\partial z}-D_{2}^{m}\theta ,$$

(14) $$\sigma_{zz}=\frac{\partial w}{\partial z}+c_{1}\frac{\partial u}{\partial x}-D_{2}^{m}\theta ,$$

(15) $$\sigma_{xz}=c_{2}\left(\frac{\partial w}{\partial x}+\frac{\partial u}{\partial z}\right),$$

in which,

(16) $$\{c_{1}\;,\;c_{2}\}=\frac{1}{\lambda +2\mu }\{\lambda \;,\;\mu \}.$$

Additionally, the motion equations and the heat conducting equation are presented by,

(17) $$\frac{\partial^{2}u}{\partial x^{2}}+c_{2}\frac{\partial^{2}u}{\partial z^{2}}+c_{3}\frac{\partial^{2}w}{\partial x\partial z}-D_{2}^{m}\frac{\partial \theta }{\partial x}=\frac{\partial^{2}u}{\partial t^{2}}-\Omega^{2}u+2\Omega \frac{\partial w}{\partial t},$$

(18) $$\frac{\partial^{2}w}{\partial z^{2}}+c_{2}\frac{\partial^{2}w}{\partial x^{2}}+c_{3}\frac{\partial^{2}u}{\partial x\partial z}-D_{2}^{m}\frac{\partial \theta }{\partial z}=\frac{\partial^{2}w}{\partial t^{2}}-\Omega^{2}w-2\Omega \frac{\partial u}{\partial t},$$

(19) $$\nabla^{2}\theta =D_{1}\frac{\partial \theta }{\partial t}+\epsilon D_{1}^{m}\frac{\partial e}{\partial t},$$

in which,

(20) $$c_{3}=c_{1}+c_{2},\;\;\;\epsilon =\frac{\gamma^{2}T_{0}}{\rho c_{E}\left(\lambda +2\mu \right)}.$$

## 4. Solution of the Problem

The following expressions can be used to describe physical variables in terms of normal modes.

(21) $$\left\{u,w,\theta ,\sigma_{ij}\right\}\left(x,z,t\right)=\left\{u^{*},w^{*},\theta^{*},\sigma_{ij}^{*}\right\}\left(z\right)e^{𝒾k\left(x-ct\right)},$$

where $𝒾=\sqrt{-1}$, c represents the velocity of phase, k is the wave number across the x-direction, $u^{*}$, $w^{*}$, $\theta^{*}$ and $\sigma_{ij}^{*}$ are the field amplitudes quantities.

Replacing Equations (21) in Equations (17)–(19), we have,

(22) $$c_{2}\frac{d^{2}u^{*}}{dz^{2}}-c_{4}u^{*}+𝒾kc_{3}\frac{dw^{*}}{dz}+c_{7}w^{*}-𝒾k{\bar{D}}_{2}^{m}\theta^{*}=0,$$

(23) $$\frac{d^{2}w^{*}}{dz^{2}}-c_{5}w^{*}+𝒾kc_{3}\frac{du^{*}}{dz}-c_{7}u^{*}-{\bar{D}}_{2}^{m}\frac{d\theta^{*}}{dz}=0,$$

(24) $$\frac{d^{2}\theta^{*}}{dz^{2}}-c_{6}\theta^{*}+𝒾kc_{8}u^{*}+c_{8}\frac{dw^{*}}{dz}=0,$$

where,

(25) $$\begin{array}{c} c_{4}=k^{2}\left(1-c^{2}\right)-\Omega^{2},\;\;\;c_{5}=k^{2}\left(c_{2}-c^{2}\right)-\Omega^{2},\\ c_{6}=k\left(k-𝒾c{\bar{D}}_{1}\right),\;\;\;c_{7}=2𝒾ck\Omega ,\;\;\;c_{8}=𝒾ck\epsilon {\bar{D}}_{1}^{m}, \end{array}$$

in which,

(26) $$\begin{array}{c} {\bar{D}}_{1}=1+\overset{N}{\underset{n=1}{\sum }}\tau_{1}^{n}{\left(-𝒾ck\right)}^{n}\\ {\bar{D}}_{1}^{m}=1+\delta_{1m}\overset{N}{\underset{n=1}{\sum }}\tau_{1}^{n}{\left(-𝒾ck\right)}^{n}\\ {\bar{D}}_{2}^{m}=1+\delta_{2m}\overset{N}{\underset{n=1}{\sum }}\tau_{2}^{n}{\left(-𝒾ck\right)}^{n} \end{array}\},\;\;\;N\geq 1.$$

Furthermore, the components of stress could be modified as follows:

(27) $$\sigma_{xx}^{*}=𝒾ku^{*}+c_{1}\frac{dw^{*}}{dz}-D_{2}^{m}\theta^{*},$$

(28) $$\sigma_{zz}^{*}=\frac{dw^{*}}{dz}+𝒾kc_{1}u^{*}-D_{2}^{m}\theta^{*},$$

(29) $$\sigma_{xz}^{*}=c_{2}(𝒾kw^{*}+\frac{du^{*}}{dz}).$$

The following integrated form may be obtained by resolving the differential equations system (22)–(24).

(30) $$\left(\frac{d^{6}}{dz^{6}}-C_{2}\frac{d^{4}}{dz^{4}}+C_{1}\frac{d^{2}}{dz^{2}}-C_{0}\right)\left\{u^{*},w^{*},\theta^{*}\right\}=0,$$

in which the coefficients $C_{i}$, i=0,1,2 are expressed as,

(31) $$\begin{array}{c} C_{2}=c_{5}+c_{6}-c_{8}{\bar{D}}_{2}^{m}+\frac{c_{4}-c_{3}^{2}k^{2}}{c_{2}},\\ C_{1}=\frac{1}{c_{2}}[\left(c_{5}+c_{6}-c_{8}{\bar{D}}_{2}^{m}\right)c_{4}+\left(c_{2}c_{5}-c_{3}^{2}k^{2}\right)c_{6}+c_{8}k^{2}{\bar{D}}_{2}^{m}\left(2c_{3}-1\right)+c_{7}^{2}],\\ C_{0}=\frac{1}{c_{2}}[\left(c_{4}c_{5}+c_{7}^{2}\right)c_{6}-k^{2}c_{5}c_{8}{\bar{D}}_{2}^{m}]. \end{array}$$

When $\zeta_{i},$ (i=1,2,3) is introduced into Equation (24), the result is,

(32) $$\left(\frac{d^{2}}{dz^{2}}-\zeta_{1}^{2}\right)\left(\frac{d^{2}}{dz^{2}}-\zeta_{2}^{2}\right)\left(\frac{d^{2}}{dz^{2}}-\zeta_{3}^{2}\right)\left\{u^{*},w^{*},\theta^{*}\right\}=0,$$

in which $\zeta_{i}^{2}$ represent the roots of,

(33) $$\zeta^{6}-C_{2}\zeta^{4}+C_{1}\zeta^{2}-C_{0}=0,$$

including,

(34) $$\begin{array}{c} \zeta_{1}^{2}=\frac{4C_{2}C_{3}+4\left(1-𝒾\sqrt{3}\right)\left(3C_{1}-C_{2}^{2}\right)-\left(1+𝒾\sqrt{3}\right)C_{3}^{2}}{12C_{3}},\\ \zeta_{2}^{2}=\frac{4C_{2}C_{3}+4\left(1+𝒾\sqrt{3}\right)\left(3C_{1}-C_{2}^{2}\right)-\left(1-𝒾\sqrt{3}\right)C_{3}^{2}}{12C_{3}},\\ \zeta_{3}^{2}=\frac{{\left(C_{2}+C_{3}\right)}^{2}+3\left(C_{2}^{2}-4C_{1}\right)}{6C_{3}}, \end{array}$$

wherein,

(35) $$\begin{array}{c} C_{3}^{3}=108C_{0}-36C_{1}C_{2}+8C_{2}^{3}+12C_{4},\\ C_{4}^{2}=3C_{0}\left(27C_{0}+4C_{2}^{3}-18C_{1}C_{2}\right)+3C_{1}^{2}(4C_{1}-C_{2}^{2}). \end{array}$$

The solution of Equation (30) can be obtained under the regularity criteria: $u^{*},w^{*},\theta^{*}\to 0$ as x→∞. As a result, its indefinitely bounded general solution is offered by,

(36) $$\left\{u^{*},w^{*},\theta^{*}\right\}=\overset{3}{\underset{j=1}{\sum }}\left\{1,{\bar{\zeta }}_{j},{\hat{\zeta }}_{j}\right\}A_{j}e^{-\zeta_{j}z},$$

in which $A_{j}$ (j=1,2,3) represent some parameters in terms of k, c; and ${\bar{\zeta }}_{j}$, ${\hat{\zeta }}_{j}$ indicate some distinct parameters associated with $A_{j}$. It must be mentioned that three terms having exponentials of growing in nature in the space variables x were omitted in the present approach. The following results are given by inserting Equation (36) into Equations (22) and (23)

(37) $${\bar{\zeta }}_{j}=\frac{-𝒾c_{2}\zeta_{j}^{3}+𝒾\left(c_{4}-c_{3}k^{2}\right)\zeta_{j}-c_{7}k}{k\left(c_{3}-1\right)\zeta_{j}^{2}+𝒾c_{7}\zeta_{j}+c_{5}k},\;\;\;{\hat{\zeta }}_{j}=\frac{𝒾\left[c_{2}\xi_{j}^{4}-\left(c_{4}+c_{2}c_{5}-c_{3}^{2}k^{2}\right)\xi_{j}^{2}+c_{4}c_{5}+c_{7}^{2}\right]}{\left[k\left(c_{3}-1\right)\zeta_{j}^{2}+𝒾c_{7}\zeta_{j}+c_{5}k\right]{\bar{D}}_{2}^{m}}.$$

As a result, the displacements and temperature may be written in the final equations as,

(38) $$\left\{u,w,\theta \right\}=\sum_{j=1}^{3}\left\{1,{\bar{\zeta }}_{j},{\hat{\zeta }}_{j}\right\}A_{j}e^{𝒾k\left(x-ct\right)-\zeta_{j}z}.$$

Furthermore, the final expressions of stresses may be derived using the previous equations and Equations (27)–(29). That is,

(39) $$\left\{\sigma_{xx},\sigma_{zz},\sigma_{xz}\right\}=\sum_{j=1}^{3}\left\{\xi_{j},{\bar{\xi }}_{j},{\hat{\xi }}_{j}\right\}A_{j}e^{𝒾k\left(x-ct\right)-\zeta_{j}z},$$

in which,

(40) $$\xi_{j}=𝒾k-c_{1}\zeta_{j}{\bar{\zeta }}_{j}-{\bar{D}}_{2}^{m}{\hat{\zeta }}_{j},\;\;\;{\bar{\xi }}_{j}=𝒾c_{1}k-\zeta_{j}{\bar{\zeta }}_{j}-{\bar{D}}_{2}^{m}{\hat{\zeta }}_{j},\;\;\;{\hat{\xi }}_{j}=c_{2}(𝒾k{\bar{\zeta }}_{j}-\zeta_{j}).$$

## 5. Boundary Conditions

Along with the regularity criteria of the solution at infinity employed in Equation (36) to obtain displacements and temperatures in terms of arbitrary parameters $A_{j}$, (j=1,2,3). The following boundary criteria must be used to derive such parameters. The present half-space may be exposed to a time-dependent thermal shock and traction-free surface as a first case. In addition, one can consider the second case in which the surface of the half-space is firmly fixed and a thermal shock is exposed to it.

### 5.1. Mechanical Conditions

Two cases of mechanical boundary conditions are considered. The boundary of the half-space z=0 in the first case is traction free, i.e.,

(41) $$\sigma_{xz}\left(x,0,t\right)=0,\;\;\;\sigma_{zz}\left(x,0,t\right)=0.$$

### 5.2. Thermal Condition

In addition to the above two cases, the surface of the half-space is exposed to a time-dependent thermal shock. As the magnitude of the thermal shock wave is not entirely fixed but decaying over time, we must consider,

(42) $$\theta \left(x,0,t\right)=\Theta e^{𝒾k\left(x-ct\right)},$$

where $\Theta =\theta_{0}e^{-bt}$ in which $\theta_{0}$ is a constant and in particular, if the value of the decaying parameter b is taken to be zero we will obtain a thermal shock waveform with constant magnitude [41].

### 5.3. Traction Free Half-Space

Using the expressions of the stresses $\sigma_{xz}$, $\sigma_{zz}$ and the temperature θ into the above first case of boundary conditions, we can determine the following equations satisfied with the parameters:

(43) $$\left\{\begin{array}{c} A_{1}\\ A_{2}\\ A_{3} \end{array}\right\}={\left[\begin{array}{ccc} {\bar{\xi }}_{1} & {\bar{\xi }}_{2} & {\bar{\xi }}_{3}\\ {\hat{\xi }}_{1} & {\hat{\xi }}_{2} & {\hat{\xi }}_{3}\\ {\hat{\zeta }}_{1} & {\hat{\zeta }}_{2} & {\hat{\zeta }}_{3} \end{array}\right]}^{-1}\left\{\begin{array}{c} 0\\ 0\\ \Theta \end{array}\right\}.$$

Therefore, each field’s quantities such as temperature, displacement, and stresses will be simply given for both cases of boundary conditions.

## 6. Special and Particular Cases

We examine some unique and particular cases for different values of the parameters applied to the problem, such as $\tau_{1}$, $\tau_{2}$, m, N, and Ω.

### 6.1. A Special Case

The most important special case is that the above discussion may be converted to an isotropic medium without rotation effect when we set

(44) Ω=0.

### 6.2. Particular Cases

We derive several distinct findings for different limiting values of the parameters discussed in Equations (2) and (4), corresponding to various thermoelastic models. Here we discussed a refined generalized thermoelasticity theory in the context of a one/two-relaxation power-law model. The heat conduction equation presented in Equation (2) includes at least three generalized thermoelasticity theories. These theories contain the coupled thermoelasticity (CTE) theory [1], the simple Lord and Shulman (LS) thermoelasticity theory [2] as well as the simple Green and Lindsay (GL) thermoelasticity theory [3]. Therefore, one can summarize the above special cases from Equations (2) and (4) as documented here:

(a) When we put $D_{1}=D_{1}^{m}=D_{2}^{m}=1,$ we obtain the reduced equations of the CTE theory.
(b) When we put m=1, N=1 and $\tau_{1}\geq 0,$ we achieve the equations of the simple LS theory.
(c) The simple GL theory is obtained by putting m=2, N=1 and $\tau_{2}\geq \tau_{1}\geq 0$.
(d) If we put m=1, N>1 and $\tau_{1}\geq 0,$ we achieve the equations of the refined LS theory.
(e) Finally, if we put m=2, N>1 and $\tau_{2}\geq \tau_{1}\geq 0,$ we obtain the refined GL theory.

## 7. Numerical Results and Discussions

Some numerically computed findings have been illustrated in this section. The physical parameters utilized in the computations are as follows at $T_{0}=273\;K$ [42]:


(45) $$\begin{array}{c} \lambda =7.76\times 10^{9}{\;N\;m}^{-2},\;\;\;\mu =3.86\times 10^{10}{\;N\;m}^{-2},\;\;\;\rho =8954{\;\mathrm{kg}\;m}^{-3},\\ k=150{\;W\;m}^{-1}K^{-1},\;\;\;c_{E}=383.1{\;J\;\mathrm{kg}}^{-1}{\;K}^{-1},\;\;\;\alpha_{t}=3.78\times 10^{-4}\;K^{-1}. \end{array}$$

Except as otherwise specified, numerical results are derived for t=0.5; $\theta_{0}=10.5$; $\tau_{1}=0.3$; $\tau_{2}=0.35$; b=0.3; and Ω=0.5.

The time t=0.5 is fixed in all plots while the rotation factor Ω and decaying parameter b should have distinct values to show their effects on all variables of the rotating half-space. Firstly, 12 2D figures are presented to show the effect of Ω and b on the field quantities. In the 2D figures, the outcomes of the CTE theory are compared with those of the simple and refined LS theory on one hand and with those of the simple and refined GL theory on the other hand. Next, six 3D figures are presented for fixed values of the rotation factor Ω=0.5 and the decaying parameter b=0.3. In such figures, the outcomes of the CTE theory are compared with those of the refined LS and GL theories. The outcomes of CTE theory are presented in all figures. Accordingly, the results of the simple LS and GL theories (LS (s) and GL (s)) are given when N=1 while the corresponding results of the refined LS and GL theories (LS (r) and GL (r)) are given when N=5. The 2D results are shown across the z-axis with x=1 for temperature and stresses while z=3 for the displacements.

**Figure 1:**
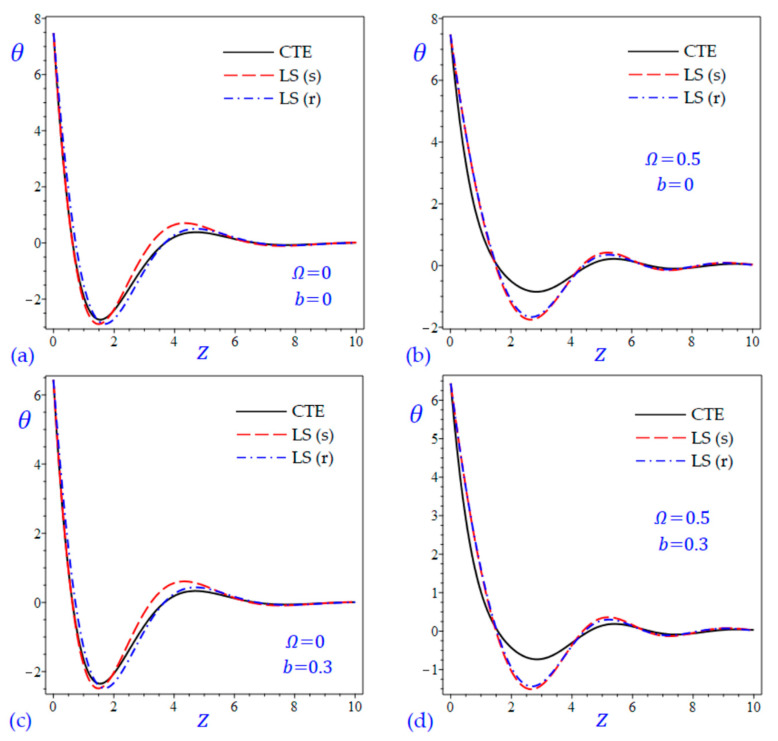
The effect of rotation factor Ω and decaying parameter b on the temperature θ of the rotating half-space using CTE and LS theories: (**a**) Ω=0, b=0, (**b**) Ω=0.5, b=0, (**c**) Ω=0, b=0.3, and (**d**) Ω=0.5, b=0.3.

**Figure 2:**
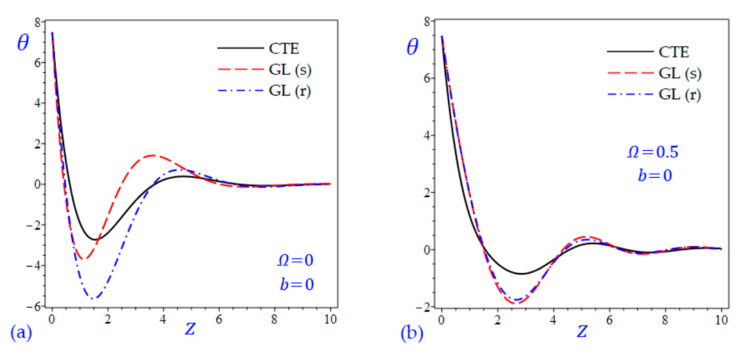

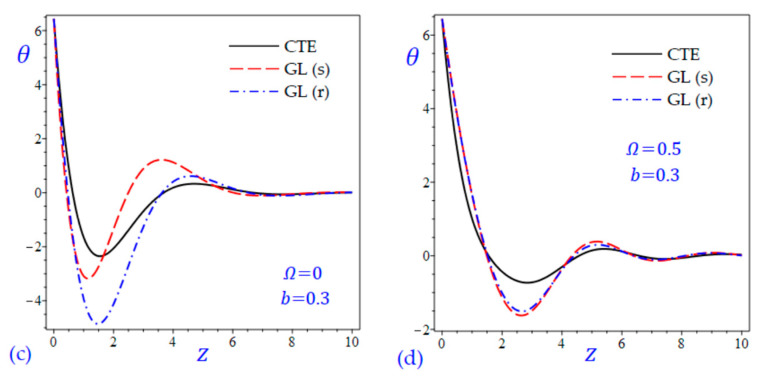
The effect of rotation factor Ω and decaying parameter b on the temperature θ of the rotating half-space using CTE and GL theories: (**a**) Ω=0, b=0, (**b**) Ω=0.5, b=0, (**c**) Ω=0, b=0.3, and (**d**) Ω=0.5, b=0.3.

Figure 1 shows the temperature of the rotating half-space using CTE theory, simple (N=1) Lord and Shulman theory (LS (s)), and refined (N=5) Lord and Shulman theory (LS (r)). Distinct values of Ω and b are used. Similar results are presented in Figure 2 to show the temperature of the rotating half-space using CTE theory, simple (N=1) Green and Lindsay theory (GL (s)), and refined (N=5) Green and Lindsay (GL (r)). It is clear that the inclusion of the rotation factor Ω causes an increase in the wavelength and an increase in the amplitude of the temperature wave. However, the inclusion of the decaying parameter b causes a sharp increase in the wave amplitude. In addition, the wave of the temperature θ of CTE has the smallest amplitude compared with the other waves due to the simple and refined theories. This is more obvious when the rotation is included as shown in Figure 1. Without including the rotation, the temperature due to simple and refined GL theories are distinct as shown in Figure 2a,c. and this is irrespective of the value of the decaying parameter b.

**Figure 3:**
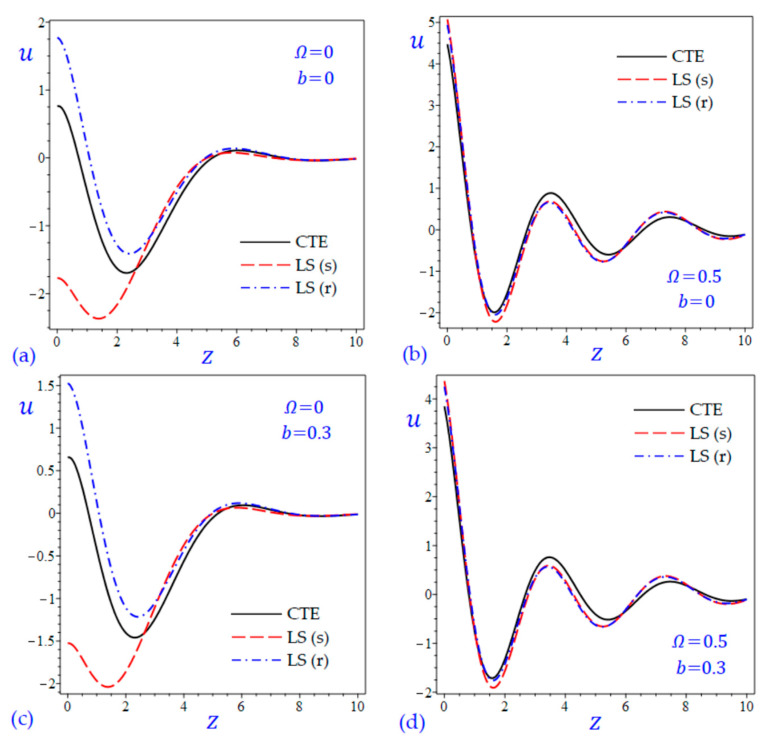
The effect of rotation factor Ω and decaying parameter b on the horizontal displacement u of the rotating half-space using CTE and LS theories: (**a**) Ω=0, b=0, (**b**) Ω=0.5, b=0, (**c**) Ω=0, b=0.3, and (**d**) Ω=0.5, b=0.3.

**Figure 4:**
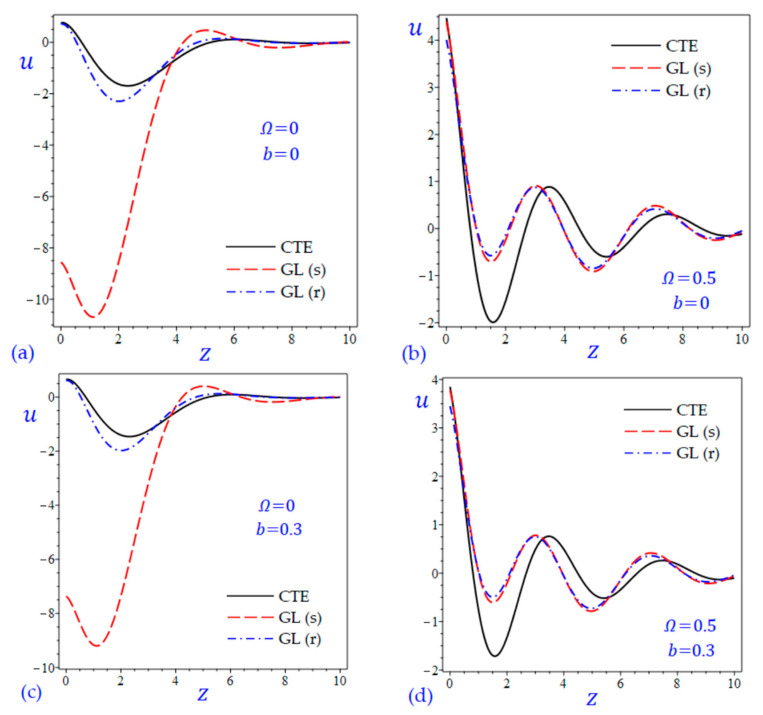
The effect of rotation factor Ω and decaying parameter b on the horizontal displacement u of the rotating half-space using CTE and GL theories: (**a**) Ω=0, b=0, (**b**) Ω=0.5, b=0, (**c**) Ω=0, b=0.3, and (**d**) Ω=0.5, b=0.3.

Figure 3 and Figure 4 show the horizontal displacement u of all theories. The waves of the horizontal displacement may have the same behavior when Ω=0.5. In the interval 0≤z<2.4 in Figure 3a,c without rotation, the horizontal displacement u due to the CTE theory may be the upper limits of those due to the LS (s) theory, and the lower limits of those due to the LS (r) theory. However, the horizontal displacement u due to the CTE theory are the upper limits of those due to the GL (s) and GL (r) theories as shown in Figure 4a,c.

**Figure 5:**
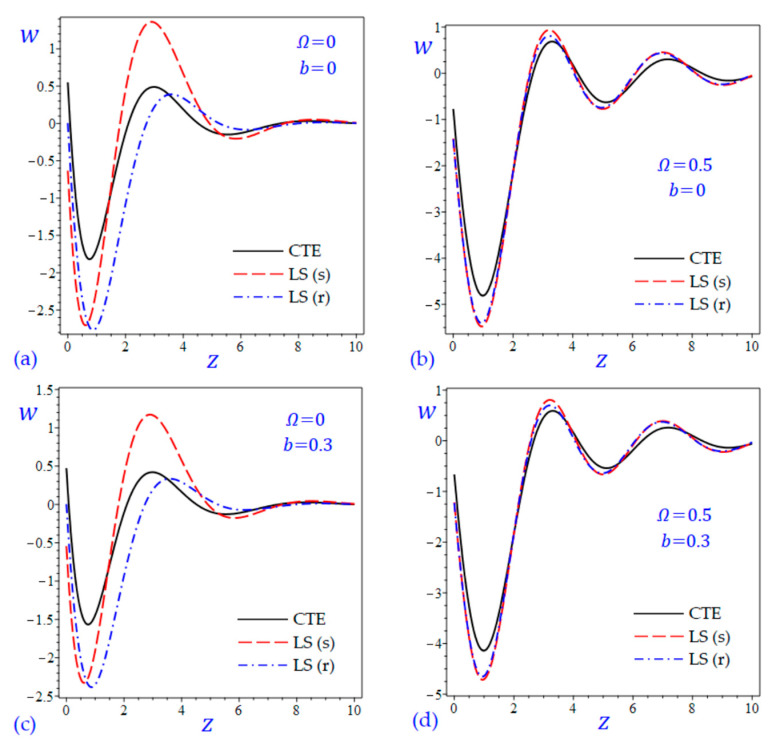
The effect of rotation factor Ω and decaying parameter b on the vertical displacement w of the rotating half-space using CTE and LS theories: (**a**) Ω=0, b=0, (**b**) Ω=0.5, b=0, (**c**) Ω=0, b=0.3, and (**d**) Ω=0.5, b=0.3.

**Figure 6:**
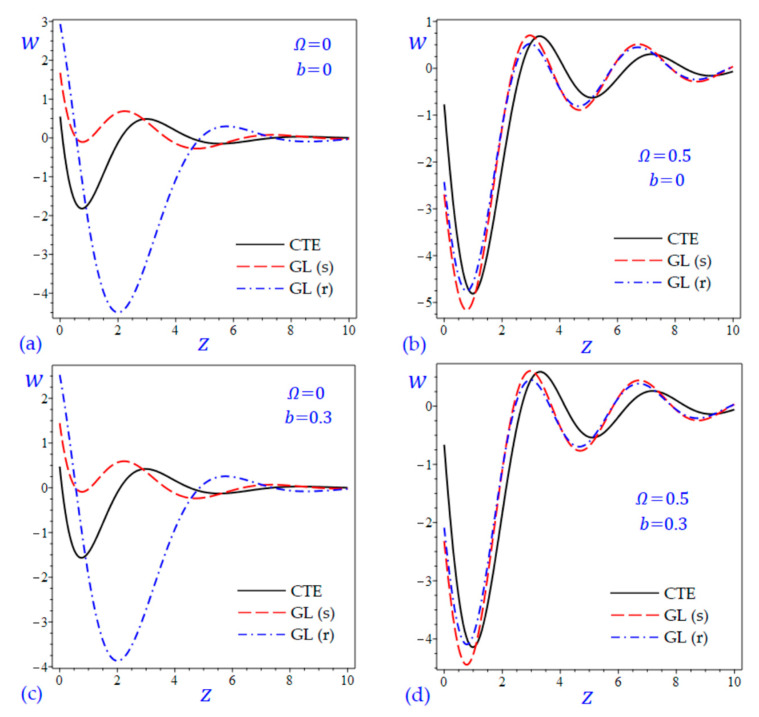
The effect of rotation factor Ω and decaying parameter b on the vertical displacement w of the rotating half-space using CTE and GL theories: (**a**) Ω=0, b=0, (**b**) Ω=0.5, b=0, (**c**) Ω=0, b=0.3, and (**d**) Ω=0.5, b=0.3.

In Figure 5 and Figure 6, the vertical displacement w of the rotating half-space is presented due to all theories. In Figure 5, the amplitude of the vertical displacement wave due to the CTE theory is the smallest one. However, in Figure 6a,c (when the rotation is omitted) the waves of the vertical displacement due to the simple Green-Lindsay (GL (s)) theory have the smallest amplitude. When the rotation of the half-space is included (see Figure 5b,d and Figure 6b,d) all theories have the same behaviors to describe the wave of vertical displacement. In such figures, the waves of w of the rotating half-space due to the refined LS and GL theories may be intermediate to those due to the CTE and simple theories. However, the refined Green-Lindsay (GL (r)) theory gives the wave of vertical displacement with high amplitude as shown in Figure 6a,c.

**Figure 7:**
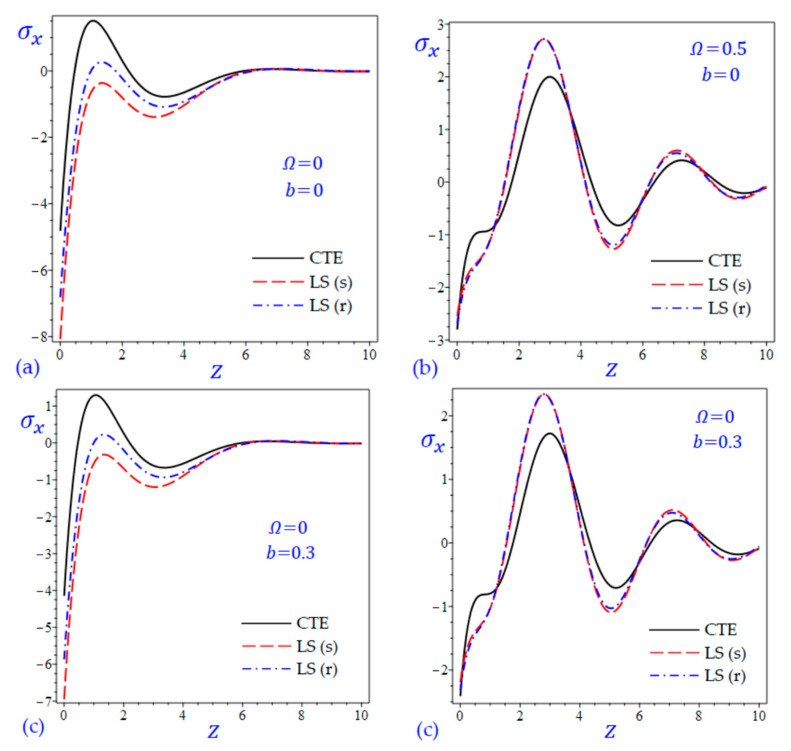
The effect of rotation factor Ω and decaying parameter b on the longitudinal normal stress $\sigma_{x}$ of the rotating half-space using CTE and LS theories: (**a**) Ω=0, b=0, (**b**) Ω=0.5, b=0, (**c**) Ω=0, b=0.3, and (**d**) Ω=0.5, b=0.3.

**Figure 8:**
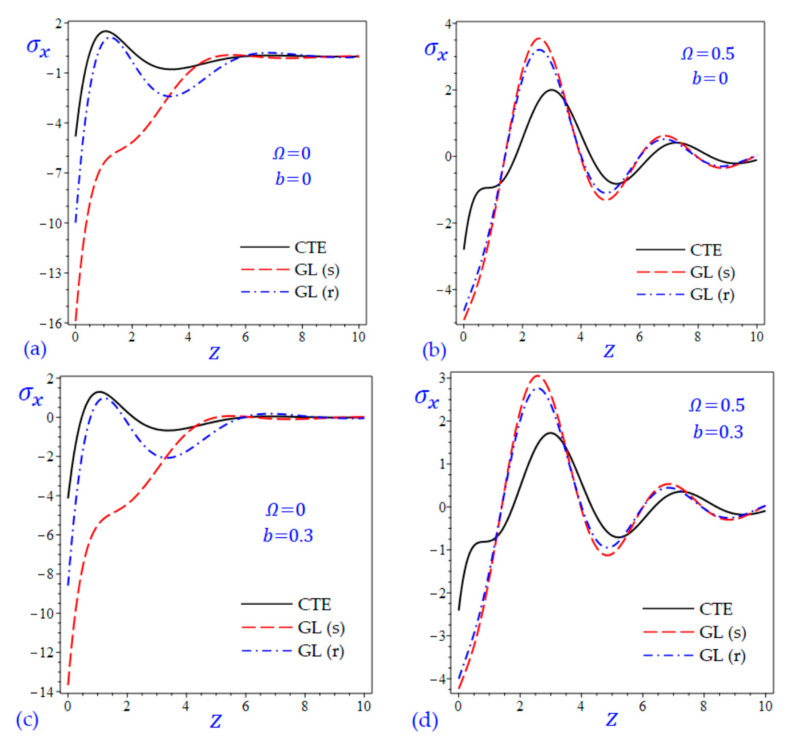
The effect of rotation factor Ω and decaying parameter b on the longitudinal normal stress $\sigma_{x}$ of the rotating half-space using CTE and GL theories: (**a**) Ω=0, b=0, (**b**) Ω=0.5, b=0, (**c**) Ω=0, b=0.3, and (**d**) Ω=0.5, b=0.3.

In Figure 7 and Figure 8, the longitudinal normal stress $\sigma_{x}$ of the rotating and non-rotating half-space have been illustrated due to all theories. For rotating half-space, the plots of $\sigma_{x}$ due to simple and refined theories (Figure 7b,d and Figure 8b,d) being very close to each other. In such figures, the number of waves may be increased. For nonrotating half-space, the plots of $\sigma_{x}$ due to LS (r) theory being intermediate those of CTE and LS (s) theories as shown in Figure 7a,c. this is the same in Figure 8a,c when z<3.2. in general, for nonrotating half-space (Figure 7a,c and Figure 8a,c), the plots of longitudinal normal stress $\sigma_{x}$ due to LS (s) and GL (s) theories having the smallest amplitude.

**Figure 9:**
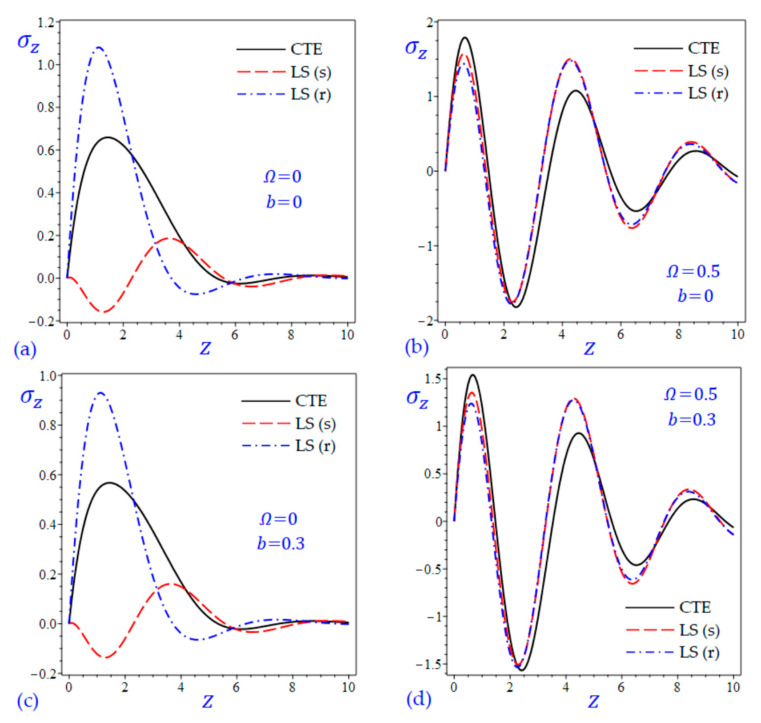
The effect of rotation factor Ω and decaying parameter b on the transverse normal stress $\sigma_{z}$ of the rotating half-space using CTE and LS theories: (**a**) Ω=0, b=0, (**b**) Ω=0.5, b=0, (**c**) Ω=0, b=0.3, and (**d**) Ω=0.5, b=0.3.

**Figure 10:**
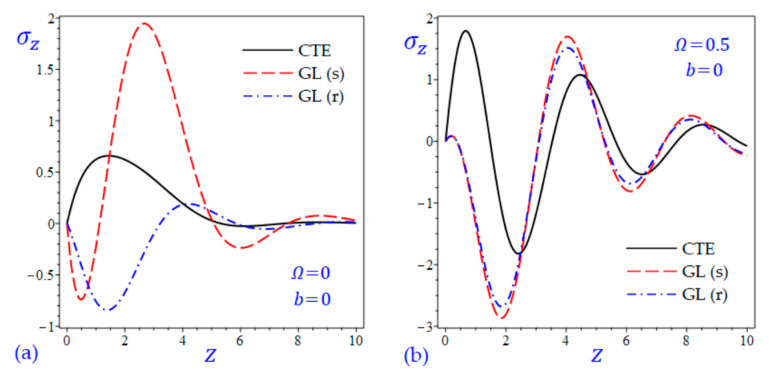

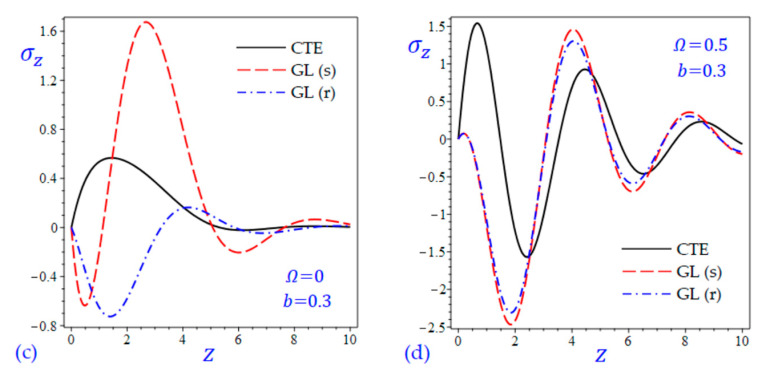
The effect of rotation factor Ω and decaying parameter b on the transverse normal stress $\sigma_{z}$ of the rotating half-space using CTE and GL theories: (**a**) Ω=0, b=0, (**b**) Ω=0.5, b=0, (**c**) Ω=0, b=0.3, and (**d**) Ω=0.5, b=0.3.

Figure 9 and Figure 10 show the transverse normal stress $\sigma_{z}$ of the rotating and non-rotating half-space due to all theories. The waves of transverse normal stress $\sigma_{z}$ of the non-rotating half-space have different wavelengths and different amplitudes according to the used theory. For rotating half-space, the LS (s) and LS(r) theories yield transverse normal stress $\sigma_{z}$ close to each other and maybe close to those due to the CTE theory. Concerning the effect of decaying parameter b, the amplitudes of all outcomes decrease when the decaying parameter b is included.

**Figure 11:**
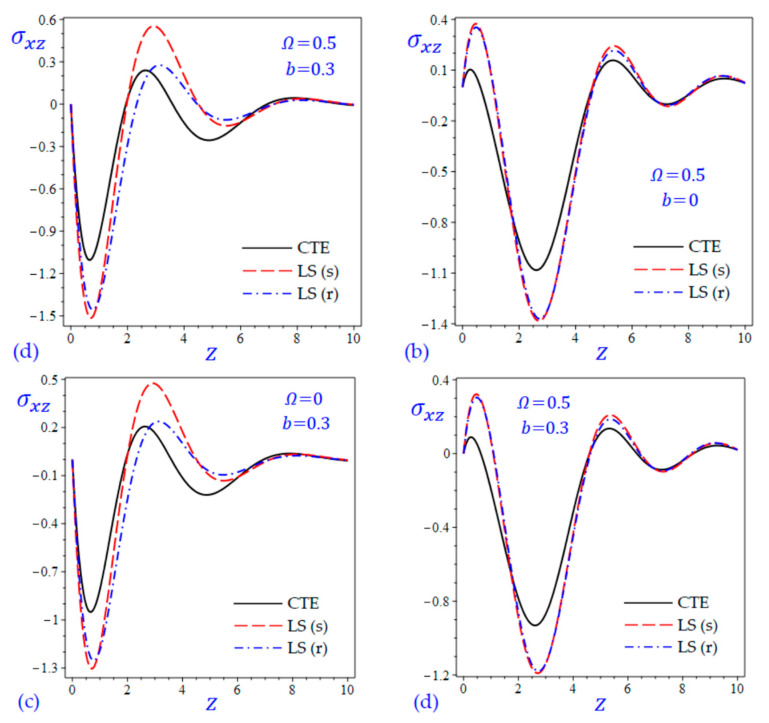
The effect of rotation factor Ω and decaying parameter b on the transverse shear stress $\sigma_{xz}$ of the rotating half-space using CTE and LS theories: (**a**) Ω=0, b=0, (**b**) Ω=0.5, b=0, (**c**) Ω=0, b=0.3, and (**d**) Ω=0.5, b=0.3.

**Figure 12:**
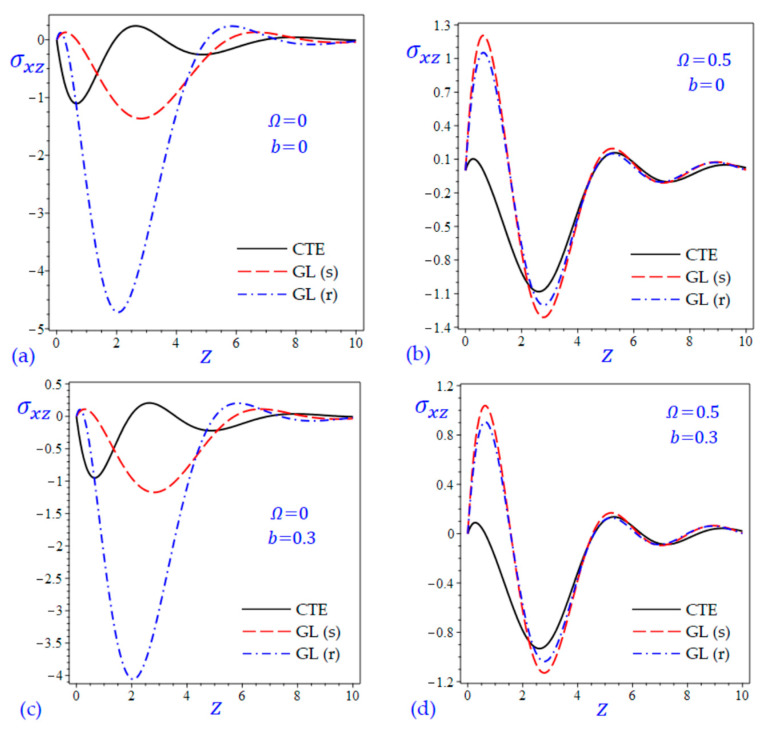
The effect of rotation factor Ω and decaying parameter b on the transverse shear stress $\sigma_{xz}$ of the rotating half-space using CTE and GL theories: (**a**) Ω=0, b=0, (**b**) Ω=0.5, b=0, (**c**) Ω=0, b=0.3, and (**d**) Ω=0.5, b=0.3.

Figure 11 and Figure 12 show the transverse shear stress $\sigma_{xz}$ of the rotating and non-rotating half-space due to all theories. Figure 11a,c show that the LS (r) theory yields waves of transverse shear stress intermediate to those of the CTE and LS (s) theories. However, Figure 12a,c show that GL (r) theory yields waves of transverse shear stress with the largest amplitude compared with those of the CTE and GL (s) theories.

**Figure 13:**
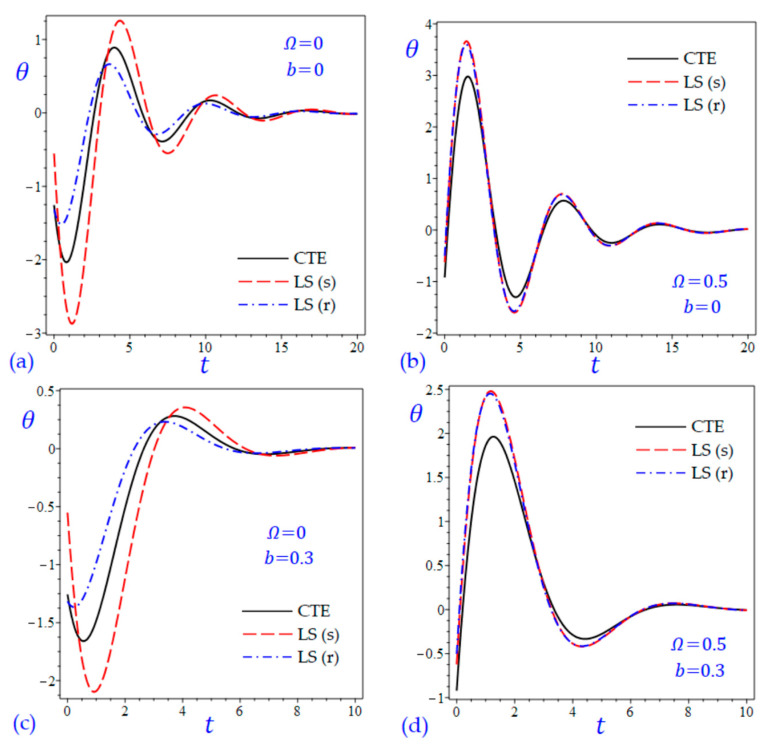
The variation of temperature θ versus the dimensionless time t considering the effect of rotation factor Ω and decaying parameter b using CTE and LS theories: (**a**) Ω=0, b=0, (**b**) Ω=0.5, b=0, (**c**) Ω=0, b=0.3, and (**d**) Ω=0.5, b=0.3.

**Figure 14:**
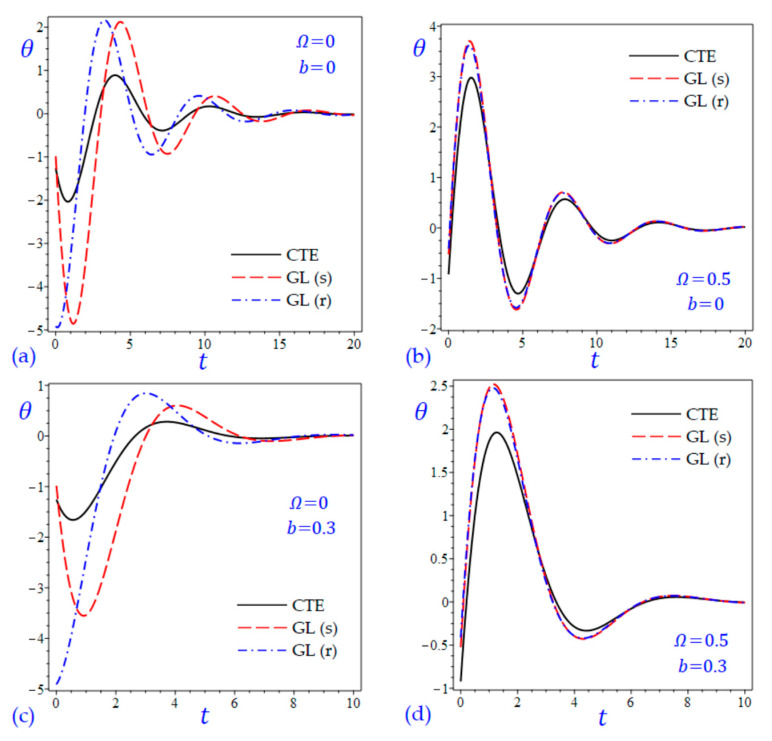
The variation of temperature θ versus the dimensionless time t considering the effect of rotation factor Ω and decaying parameter b using CTE and GL theories: (**a**) Ω=0, b=0, (**b**) Ω=0.5, b=0, (**c**) Ω=0, b=0.3, and (**d**) Ω=0.5, b=0.3.

For the sake of completeness, two additional figures are added to discuss the behavior of temperature versus the dimensionless time t. Figure 13 and Figure 14 show the temperature θ of the half-space versus the time *t* due to all theories. Both x and z are fixed to be *x* = *z* = 1. It is interesting to see that the temperature may be changed its sign from negative to positive or vice versa according to the period of dimensionless time. In all cases, the wave of the temperature θ may be reduced as t increases and will vanish for high values of the dimensionless time t. As we discussed before, the inclusion of the rotation factor Ω causes an increase in the amplitude of the temperature wave (Figure 13b,d and Figure 14b,d). In addition, the wave of the temperature θ of CTE has the smallest amplitude compared with the other waves due to the simple and refined theories. However, the inclusion of the decaying parameter b causes a sharp increase in the wave amplitude and a decrease in the wavelength (Figure 13c,d and Figure 14c,d). This is more obvious when the rotation is included. Without including the rotation, the temperature due to simple and refined GL theories are distinct as shown in Figure 13a,c and Figure 14a,c and this is irrespective of the value of the decaying parameter b. In these figures, the waves of θ of the non-rotating half-space due to the CTE theory may be intermediate to those due to the simple and refined LS theories as shown in Figure 13a,c; and intermediate to those due to the simple and refined GL theories in Figure 14a,c.

**Figure 15:**
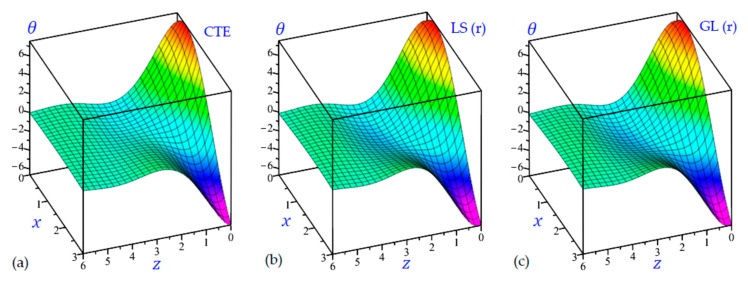
3D distributions of the temperature θ of the rotating half-space using: (**a**) CTE theory, (**b**) LS (r) theory, and (**c**) GL (r) theory (Ω=0.5, b=0.3).

It is interesting now to present some 3D plots of the field variables. The half-space is rotating with Ω=0.5 and the effect of decaying parameter b is included with b=0.3. All outcomes approach zero for high values of z. Figure 15 shows the temperature θ of the rotating half-space due to the CTE, LS (r), and GL (r) theories. The smallest temperature occurs at z=0 and x=3 while the maximum value of θ occurs at z=0 and x=0.3.

**Figure 16:**
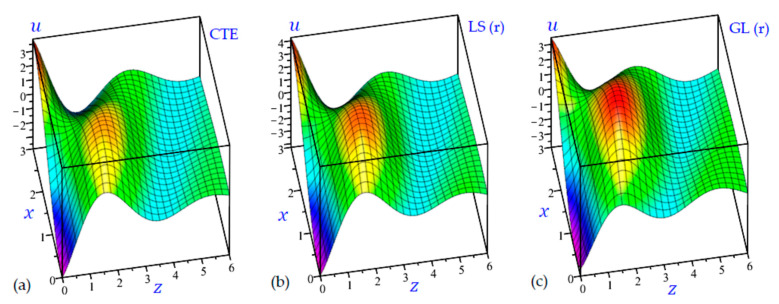
3D distributions of the horizontal displacement u of the rotating half-space using: (**a**) CTE theory, (**b**) LS (r) theory, and (**c**) GL (r) theory (Ω=0.5, b=0.3).

Figure 16 shows the horizontal displacement u of the rotating half-space with decaying parameter b=0.3 due to all theories along with the z-axis. The amplitude of the horizontal displacement wave due to CTE theory is the smallest one. However, all theories give the displacement u with the same behavior. The LS (r) theory yields the heist horizontal displacement u.

**Figure 17:**
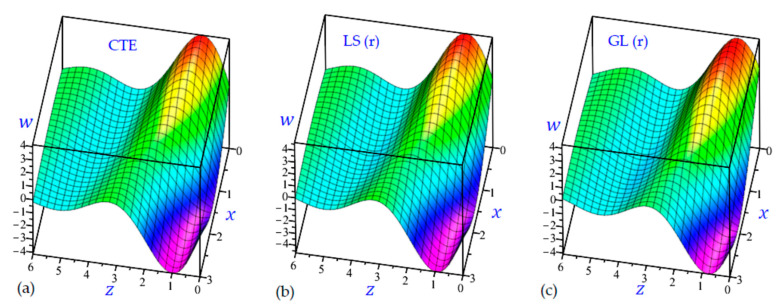
3D distributions of the transverse displacement w of the rotating half-space using: (**a**) CTE theory, (**b**) LS (r) theory, and (**c**) GL (r) theory (Ω=0.5, b=0.3).

Figure 17 shows the vertical displacement w of the rotating half-space with decaying parameter (b=0.3) due to all theories along with the z-axis. It is interesting to show that the maximum and minimum values of w occur at z=1. The maximum occurs when x=0 while the minimum occurs when x=3.

**Figure 18:**
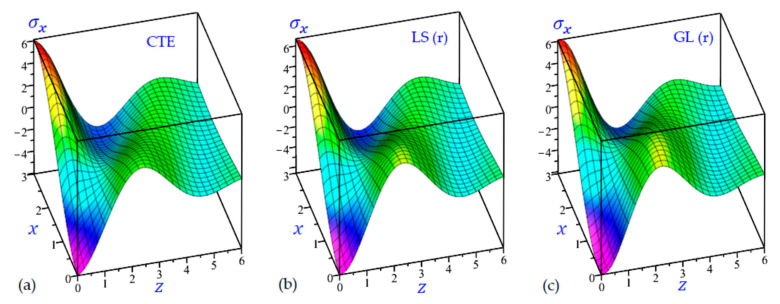
3D distributions of the longitudinal normal stress $\sigma_{x}$ of the rotating half-space using: (**a**) CTE theory, (**b**) LS (r) theory, and (**c**) GL (r) theory (Ω=0.5, b=0.3).

Figure 18 illustrates the longitudinal normal stress $\sigma_{x}$ of the rotating half-space with b=0.3 matching all CTE, LS (r), and GL (r) thermoelasticity theories. The minimum values of $\sigma_{x}$ are occurring at the origin (0, 0) while the maximum values of $\sigma_{x}$ are occurring at (0, 3). The longitudinal normal stress $\sigma_{x}$ may rapidly vanish when z→6 for the rotating half-space with (b=0.3).

**Figure 19:**
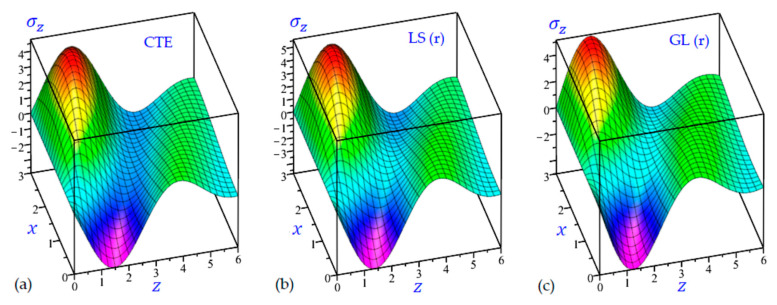
3D distributions of the transverse normal stress $\sigma_{z}$ of the rotating half-space using: (**a**) CTE theory, (**b**) LS (r) theory, and (**c**) GL (r) theory (Ω=0.5, b=0.3).

In Figure 19, the transverse normal stress $\sigma_{z}$ of the rotating half-space with decaying parameter (b=0) are illustrated due to all theories. The minimum values of $\sigma_{z}$ are occurring at (0, 1.4) while the maximum values of $\sigma_{z}$ are occurring at (3, 1). The transverse normal stress $\sigma_{x}$ starts at zero value (during the boundary conditions) and may rapidly vanish when z→6 for the rotating half-space with (b=0.3).

**Figure 20:**
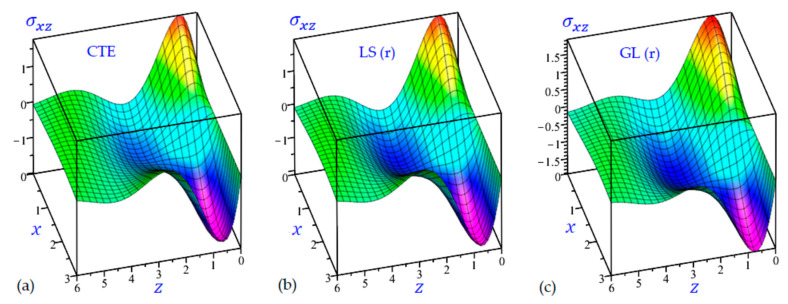
3D distributions of the transverse shear stress $\sigma_{xz}$ of the rotating half-space using: (**a**) CTE theory, (**b**) LS (r) theory, and (**c**) GL (r) theory (Ω=0.5, b=0.3).

Figure 20 illustrates the transverse shear stress $\sigma_{xz}$ of the rotating half-space with the decaying parameter matching all CTE, LS (r), and GL (r) thermoelasticity theories. The transverse shear stress $\sigma_{xz}$ is already vanished at z=0 according to the boundary condition and may tend to zero once again when z approaches six. The maximum values of $\sigma_{xz}$ are occurring when x=3, z=0.6 while the minimum values are occurring when x=0, z=0.6.

## 8. Conclusions

In this study, a unified form of refined thermoelasticity theory contains two familiar generalized thermoelasticity: Lord–Shulman theory, and Green–Lindsay theory. The classical coupled thermoelasticity theories as well as the simple forms of Lord–Shulman, and Green–Lindsay theories can be obtained from the unified form. Through the present unified thermoelasticity theory, one can acquire the field quantities of a rotating/non-rotating half-space with and without the effect of the decay parameter. Such medium is subjected to a time-dependent thermal shock taking into account that the magnitude of the thermal shock wave is not purely constant but decaying over time. A special case of a thermal shock waveform with constant magnitude is also considered.

The temperature, displacements, and stresses of the present problem are analytically obtained. Some plots of these field variables are presented in two- and three-dimensional illustrations in the context of refined theories. The inclusion of rotation and/or the decaying over time of the thermal shock wave are both considered. Many examples and applications are given to compare the results of all theories, regardless of whether or not the rotating half-space is subject to decay over time. Additional plots of temperature versus the dimensionless time are considered. As long as the refined theories (LS (r) and GL (r)) are used, the outcomes are more accurate. The inclusion of the decaying parameter causes a sharp increase in the wave amplitude of most variables. Such waves due to the CTE theory have the smallest amplitude compared with the other waves due to the simple and refined theories. Without including the rotation, the field variables due to simple and refined GL theories are distinct and this is irrespective of the value of the decaying parameter. For a rotating half-space, the LS (s) and LS (r) theories yield outcomes very close to each other and maybe close to those due to the CTE theory. In general, the present work is useful and valuable for the analysis of different problems.

## Data Availability

Not applicable.
